# Cetaceans evolution: insights from the genome sequences of common minke whales

**DOI:** 10.1186/s12864-015-1213-1

**Published:** 2015-01-22

**Authors:** Jung Youn Park, Yong-Rock An, Naohisa Kanda, Chul-Min An, Hye Suck An, Jung-Ha Kang, Eun Mi Kim, Du-Hae An, Hojin Jung, Myunghee Joung, Myung Hum Park, Sook Hee Yoon, Bo-Young Lee, Taeheon Lee, Kyu-Won Kim, Won Cheoul Park, Dong Hyun Shin, Young Sub Lee, Jaemin Kim, Woori Kwak, Hyeon Jeong Kim, Young-Jun Kwon, Sunjin Moon, Yuseob Kim, David W Burt, Seoae Cho, Heebal Kim

**Affiliations:** Biotechnology Research Division, National Fisheries Research & Development Institute, Gijang gun, Busan, 619-705 Republic of Korea; Cetacean Research Institute, National Fisheries Research & Development Institute, Nam-gu, Ulsan, 680-050 Republic of Korea; The Institute of Cetacean Research, Toyomi 4-5, Chuo-ku, Tokyo 104-0055 Japan; Codes division, Insilicogen,Inc., Suwon, Gyeonggi-do 441-813 Republic of Korea; TNT Research, #924 Doosan Venture Digm, Anyang, Gyeonggi-do 431-755 Republic of Korea; Department of Agricultural Biotechnology, Seoul National University, Seoul, 151-921 Republic of Korea; Interdisciplinary Program in Bioinformatics, Seoul National University, Seoul, 151-742 Republic of Korea; C&K Genomics, Seoul National University Research Park, Seoul, 151-919 Republic of Korea; Department of Genome Sciences, University of Washington, Seattle, WA 98195-5065 USA; Department of Life Science, Ewha Womans University, Seoul, Korea; The Roslin Institute, University of Edinburgh, Midlothian, EH25 9GR UK

**Keywords:** Cetaceans, Common minke whale, Positive selection, Genome

## Abstract

**Background:**

Whales have captivated the human imagination for millennia. These incredible cetaceans are the only mammals that have adapted to life in the open oceans and have been a source of human food, fuel and tools around the globe. The transition from land to water has led to various aquatic specializations related to hairless skin and ability to regulate their body temperature in cold water.

**Results:**

We present four common minke whale (*Balaenoptera acutorostrata*) genomes with depth of ×13 ~ ×17 coverage and perform resequencing technology without a reference sequence. Our results indicated the time to the most recent common ancestors of common minke whales to be about 2.3574 (95% HPD, 1.1521 – 3.9212) million years ago. Further, we found that genes associated with epilation and tooth-development showed signatures of positive selection, supporting the morphological uniqueness of whales.

**Conclusions:**

This whole-genome sequencing offers a chance to better understand the evolutionary journey of one of the largest mammals on earth.

**Electronic supplementary material:**

The online version of this article (doi:10.1186/s12864-015-1213-1) contains supplementary material, which is available to authorized users.

## Background

Cetaceans (whales, dolphins and porpoises) are a group of secondarily adapted marine mammals with a history of transition from terrestrial to aquatic environments. Although the exact origin and evolutionary history of cetaceans remains unclear, a widely accepted view is that their terrestrial ancestors returned to the seas around 50 Mya (million years ago) and finally diversified into a group of fully aquatic mammals [1]. These include nearly 85 species that can be subdivided into two suborders, the Mysticeti (baleen whales such as right whale, blue whale, humpback whale, and minke whale) and the Odontoceti (toothed whales such as sperm whales and dolphins), which arose from a common Eocene ancestor around 34 Mya. In spite of their variation in body size, all modern cetaceans are relatively similar in shape.

Aquatic life poses numerous challenges for mammals that were originally adapted for life on land [2]. Therefore, many features that were common in land mammals have changed in the evolutionary process that led to cetaceans. Cetaceans, as a result, lack a hair coat, presumably an adaptation to reduce friction and improve locomotion, and they regulate their body temperature in energetically challenging environments for endotherms using the mechanism such as the insulating layer of adipose tissue [3,4]. Moreover, the mysticetes, compared to odontocetes, lack an adult dentition but instead acquired a novel filter feeding mechanism using baleen plates to filter feed for bulky prey, and ultimately, this key specialization, permitted the evolution of gigantic body size, a hallmark of modern baleen whales [5–7].

Recently, Yim et al. [8] reported the whole genome sequencing and *de novo* assembly of the minke whale genome that support the hypotheses regarding adaptation to hypoxic resistance, metabolism under limited oxygen conditions and the development of unique morphological traits. They used a high-depth male minke whale sequence (128× average depth of coverage) to assemble the draft genome. In addition, a high-quality draft genome and three re-sequenced genomes of baiji (Yangtze River dolphin) were reported to reveal potential molecular adaptations of cetaceans to secondary aquatic life such as a decrease in olfactory and taste receptor genes and changes in vision and hearing genes [9].

In this paper, we use novel methods to analyze resequencing data from four common minke whales to reveal important insights into their evolutionary history without the need for a reference sequence. We identified genes common to cetaceans with accelerated rates of evolution when compared with other mammals, which are likely to control cetacean specific traits.

## Results and discussion

### Genome assembly, gene prediction and variant detection

DNA from four common minke whales from the Northeast Pacific were sequenced using the Illumina HiSeq 2000 whole genome shotgun sequencing protocol. The contig information of each common minke whale sample was generated from error corrected reads using the Allpath-LG algorithm [10] and is described in Additional file 1: Table S1. One sample (S30) showed better assembly statistics in comparison to the other three samples. Considering the contigs longer than 2,000 bp, the genome assembly of the S30 sample had 262,747 contigs (maximum length: 105,339, N50 length: 10,321 bp, total residue count: 2,010,222,571) with 15,243 N bases. This covered approximately 67% of the estimated common minke whale genome of 3 Gbp. The various repeat elements of the genome (SINE, LINE, etc) identified by RepeatMasker are shown in Additional file 1: Table S2. Gene prediction results from masked genome sequences of each sample are described in Additional file 1: Table S3. Using the gene predictions based on Augustus [11] and blastp [12] searches, we were able to classify contigs from each sample into four categories (Additional file 1: Table S4). After merging, extension and a bridging process based on the S30 genome assembly and the three other samples, we created a consenus genome assembly of the common minke whale. The combined genome assembly had the same maximum length as the S30 genome assembly but the N50 length and average length were slightly increased to 10,400 bp and 7,727 bp, respectively. In addition, the genome coverage was increased from 67.0% to 73.7% with 23,031 genes from BlastP. Summary statistics of the combined common minke whale genome assembly are shown in Additional file 1: Table S5 and the repeat elements are described in Additional file 1: Table S6.

The results of short read mapping obtained using Bowtie2 [13] are shown in Additional file 1: Table S7 and Additional file 1: Figure S1. Unified genotyper detected 554,937 small InDel variants and 5,137,672 Single Nucleotide Variants (SNVs). After filtering the variants, 389,542 InDels and 3,730,122 SNVs remained (detailed filtering options described in Methods). The number of variants in each sample is shown in Additional file 1: Table S8.

### Comparison to the previous genome assembly analysis

The assembly metrics showed the smaller number of contigs (262,747 vs 278,792), shorter genome length (by 0.2 Gbp), more genes predicted (by 2,426), but similar proportion of repeat elements and raw reads realignment rate (approximately 91% on average) compared to the previous version of genome assembly.

We then performed re-sequencing analysis using the 1) reported draft of common minke whale genome [8] and 2) our assembled scaffolds as reference to call SNP genotypes of our 4 common minke whale samples to examine the concordance between two studies (Additional file 1: Table S9). The number of matched loci was 550,202 and the genotype concordance was 97.95% on average. This concordance rate may indicate that our assembly metrics are comparable to the previous study of minke whale genome with high-coverage data and various libraries.

### Evolutionary phylogenetic relationships of baleen whales

**Figure 1:**
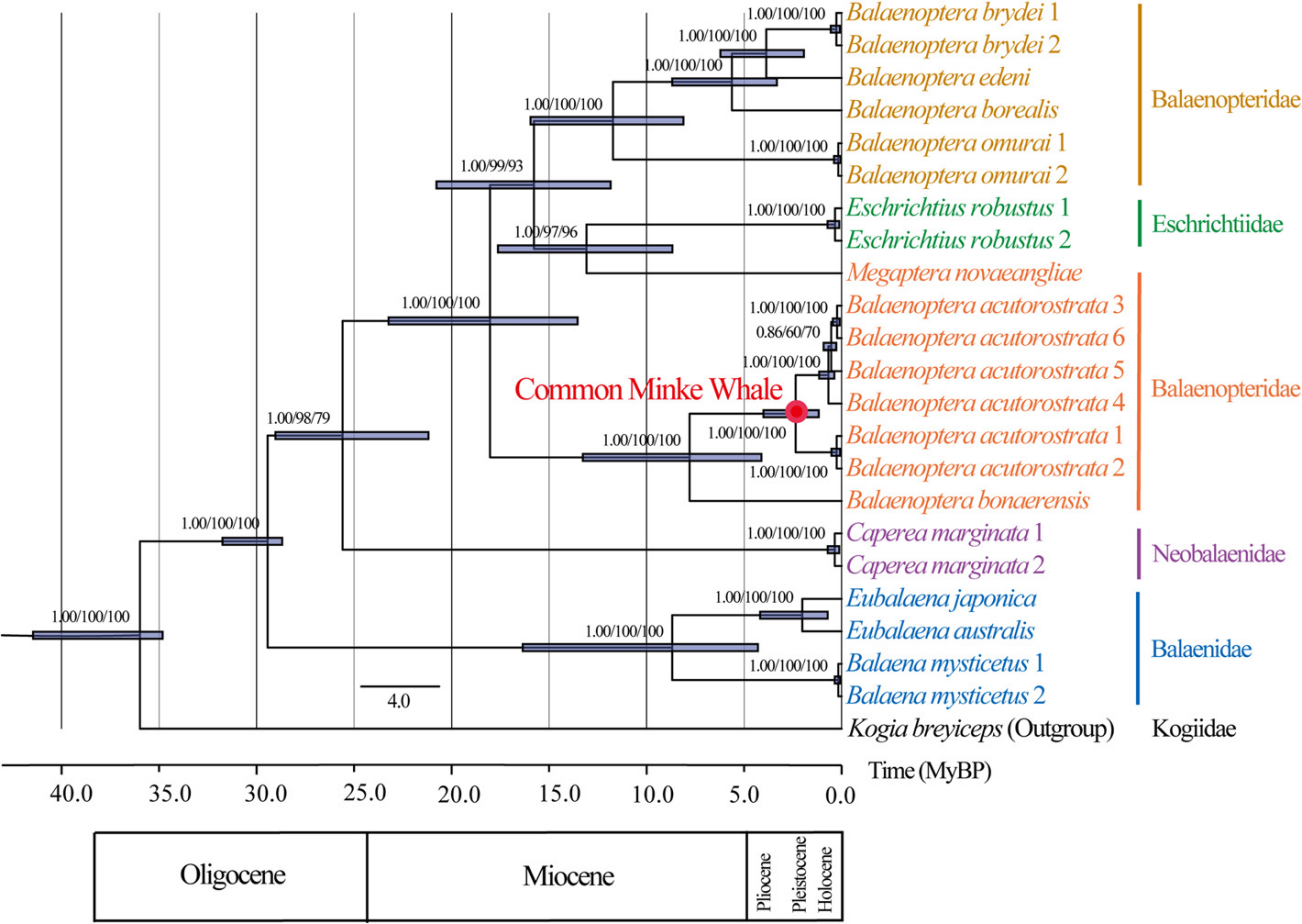
**Bayesian maximum clade credibility phylogenomic tree derived from mitochondrial genome sequences (16,435 base pairs) of 22 baleen whales.** *Kogia breviceps* (Odontoceti; Kogiidae) was used as an outgroup. The data set was also phylogenetically analyzed with BI, ML, and NJ methods and identical topology was produced. Node bars correspond to the 95% HPD for TMRCA of nodes and the numbers on the nodes represent (left to right): posterior probabilities (≥0.80) for Bayesian inference tree, bootstrap values (≥70%) for maximum likelihood tree, and bootstrap values (≥70%) for neighbor joining tree. The scale bar represents time in million years before present (MYBP).

Using four different methods (Bayesian coalescent approaches, Bayesian inference, maximum likelihood, and neighbor-joining methods), we reconstructed an evolutionary phylogenomic tree from 22 mitochondrial genome sequences consisting of 4 newly determined and 18 published sequences of the baleen whales (Figure 1 and Additional file 1: Figure S2 and Table S10). *Kogia breviceps* (Odontoceti, Kogiidae) was used as an outgroup. Common minke whales (*Balaenoptera acutorostrata*) diverged from a single maternal origin approximately 2.3574 (95% HPD, 1.1521 – 3.9212) Mya and were closely related to Antarctic minke whale (*Balaenoptera bonaerensis*). The time to the most recent common ancestor of the baleen whales was estimated to be about 28.7671 (95% HPD, 28.0336 – 31.0237).

### Genes showing accelerated evolution in the common minke whale lineage

**Figure 2:**
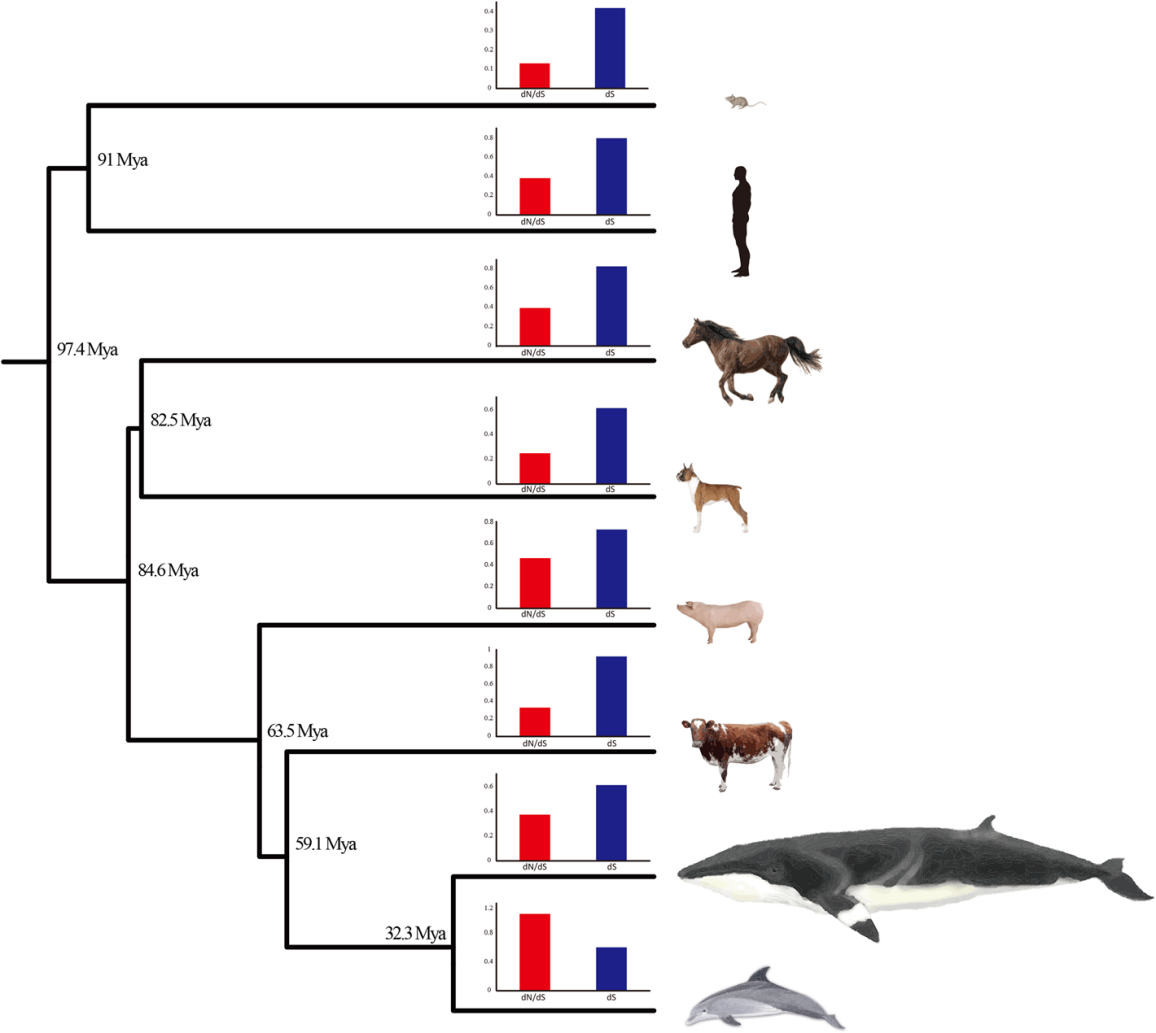
**Phylogeny of the eight mammals used in the dN/dS analysis.** The bar charts indicate the individual dN/dS (red) and dS (blue) values for each of the eight mammals. The dN/dS and dS values represent the time period of each of the eight individual lineages.

Taking into consideration the phylogenetic relationships among baleen whales, we next searched for genes that could possibly explain the specific characteristics of common minke whale. We identified 5,539 orthologous genes from 8 species (human; Homo sapiens, mouse; Mus musculus, dog; Canis familiaris, horse; Equus caballus, pig; Sus scrofa, cow; Bos taurus, dolphin; *Tursiops truncatus*, and common minke whale; *Balaenoptera acutorostrata*) to measure the rate of evolution using the ratio of nonsynonymous to synonymous substitution rate (dN/dS) analysis. Using a branch model with F3X4 codon frequencies (model = 2, NSsites = 0), we found 249 common minke whale genes that were significantly accelerated compared with the other mammal species (Figure 2). To understand the functional significance of these genes, we performed an enrichment test of Gene Ontology (GO) terms for biological processes. The genes were enriched in the function related to Wnt receptor signaling pathways (GO:0016055, P = 0.0299, 6 genes) (Additional file 1: Figure S3). The WNT gene family consists of structurally related genes that encode secreted signaling proteins. These include *WNT4*, *WNT5A*, *WNT7A*, *WNT10b*, and *WNT11*, and are important in skin development and also maintaining the hair-inducing activity of the dermal papilla [14,15]. In addition, *SFN* is a cell cycle regulator involved in epithelial keratinization and is expressed primarily in epithelial cells [16]. A mutation in this gene was found to be responsible for the repeated epilation phenotype [16,17]. These accelerated genes may have played partial roles in the evolution of skin, hair loss, and baleen plates in baleen whales. Another interesting gene, neuropeptide Y (NPY), is a neurotransmitter in the brain and ubiquitously distributed in both the central and peripheral nervous systems [18]. NPY is known to produce thermogenesis through brown adipose [19] and thus influences thermoregulation [19–21]. This gene likely reflect the physiological activities required for adaptation to underwater environment, such as cold temperature.

### Genes under positive selection during speciation of dolphins and common minke whales

We then investigated the genes under positive selection between whales and dolphins using the McDonald and Kreitman test (MKT), which is one of the most powerful and extensively used tests for detecting signatures of natural selection at the molecular level [22]. Among 11,698 orthologous genes in dolphins and common minke whales, we found evidence for significant positive selection in 13 genes (*SND1*, *ELMSAN1*, *GDI1*, *AGA*, *EIF3F*, *GLUL*, *ENSTTRT00000001243*, *USP7*, *RBL2*, *PLK1*, *HPN*, *EGR1*, and *ZNF423*). These genes reflect a paucity of nonsynonymous polymorphism relative to nonsynonymous divergence and thus is indicative of positive selection. Previously, *EGR1* was identified as one of genes differentially expressed in bowhead whales, another baleen whale species, compared to other mammals [23]. *EGR1* encodes a zinc-finger transcription factor and regulates cell growth and differentiation [24,25]. It is highly expressed during tooth development by cells of the enamel epithelium and the dental mesenchyme, and may be a key mediator of anabolic response in cementoblasts [26,27]. *RBL2* is associated with developmental delay of tooth germs and hair follicles [28]. Mutation in *AGA* causes a heritable lysosome storage disorder and is associated with changes in the facial skin, dental arches and occlusion [29,30]. Mysticeti today have baleen and lack teeth as adults, whereas Odontoceti is the clade of living toothed whales [6]. Although the roles of these genes remain incompletely understood, we speculate that this series of genes may support the transition from tooth-aided predation to filter feeding using baleen.

## Conclusions

The draft common minke whale genome sequence supports further evolutionary and comparative genomics studies of the baleen whales. The common minke whale seems to benefit from positive selection on specific genes functioning in the hairless body and thermoregulation. The presence of this benefit is supported by the detection of higher measure of adaptive evolution in protein-coding sequences.

Comparative genomics is a promising tool for determining the genetic basis of biological functions. Sequencing more mammalian genomes will facilitate our ability to annotate the human genome more thoroughly. So far, however, only a few mammals have had their complete genomes sequenced, and clearly, more data are necessary for carrying out detailed mammalian genomics. The primary limitations are due to the extreme cost and amount of time needed to sequence and assemble very large genomes that would be suitable for such analyses [31]. Despite relatively low sequencing depth, the assembled contigs and scaffolds of the common minke whale genome were sufficiently long to allow us to perform our gene prediction and comparative analyses. Our capability to generate and assemble a draft sequence for an entire mammalian genome indicates that such technology can be used to generate many other mammalian genome draft sequences in a time- and cost-effective manner.

The common minke whale genome provides unique insight into the origin and evolution of the baleen whale lineage, especially with continued refinements in the assembly and extensive functional analysis.

## Methods

### Common Minke whale genome sequencing and assembly

Four individual common minke whale samples (S30, S34, S35, and S37) were collected from the Whale Research Institute at the National Fisheries Research & Development Institute (NFRDI), Korea. The samples were caught incidentally by fishing net in Hupo, Ganggu, Pohang, and off the east coast of Korea, and were donated to NFRDI for research purposes. The process was guided and investigated by the coast guard, Cetacean Research Institute and Fisheries Cooperation Association. DNA was extracted from the muscle tissue of each common minke whale, and paired-end libraries were constructed with insert sizes of about 270 bp and 480 bp. Then 101 cycle paired-end sequencing was conducted using the Illumina HiSeq 2000 sequencer. The data are listed in Additional file 1: Table S11.

FastQC [32] was used to check the quality of the raw read data, and sequencing errors were discarded using the error-correction module of Allpaths-LG [10]. Fq2fa was used to merge error-corrected paired-end reads of each sample into one shuffled-form fasta file, with a filter option for filtering N bases in the reads. We assembled error-corrected paired-end reads using IDBA_UD [33] with the option of pre-correction and kmin = 40. Gaps (N bases) in assembled sequences were filled using Gapcloser [34] with parameter k value = 31. We carried out a genome assembly for the S30 sample using CLC Assembly with minimum contig lengths = 2000, similarity = 0.85, length fraction = 0.5, insert cost = 3, deletion cost = 3, and mismatch cost = 2.

Before gene prediction of the assembled sequence, sequence patterns including repeats were screened using RepeatMasker [35] with mammal species and the no-low, and no-is options. Augustus [11] was run across the repeat masked sequence for gene prediction, and the results were used as the input for a BLASTP [12] search. The results of the BLASTP search were filtered by length (peptides of more than 100 amino acids) and gene coverage (over 70% without gap).

The overall processes of genome assembly and gene prediction are shown in Additional file 1: Figure S4A. To maximize the gene contents of the genome assembly and construct the representative genome assembly of the four common minke whale genomes, we combined the assembly results from each sample. The process is described in further detail in Additional file 1: Figure S4B. All predicted gene sequences (>100 amino acid sequences) were merged and used as the BLASTP DB. Next, we queried the gene sequences of each sample against the DB and filtered BLASTP results (identity > 95%, 70% < q.cov and s.cov < 130%). Every contig from each sample was classified into four groups (contigs without genes, contigs with sample-specific genes, contigs with only one gene, and contigs with multiple genes). Based on the genome assembly of the S30 sample, which showed the best result from among the four samples (N50 length, N contents, coverage, maximum contig length), we added the contigs to the sample-specific genes from the other samples. We conducted multiple sequence alignments for clustered contigs from each group (contig with only one gene and contig with multiple genes) using ClustalW [36]. Contig extension and bridging were conducted based on the S30 contigs, or consensus sequence (if there was no S30 contig) in the cluster. The contig extension and bridging process is described in Additional file 1: Figure S5. The combined genome sequence was masked, and genes were predicted using the same process as in Additional file 1: Figure S4A.

The short reads were mapped to the combined assembled genome using Bowtie2 [13] with the default option. Alignment of the SAM file and removal of duplicated reads were conducted using Picard (http://picard.sourceforge.net) and SAMtools [31]. Local realignment was conducted using the Genome Analysis Toolkit (GATK) [37] and SNPs were extracted from the reads alignment file using UnifiedGenotyper, based on multi sample calling. Detected variants (QUAL < 30, QD < 5, FS > 200, MQ0 > 4, MQ0/DP > 0.1) and missing variants (which were found in one sample) were discarded from further analysis. The overall variant-calling process using GATK is shown in Additional file 1: Figure S4B.

Resequencing analysis based on the previously reported draft genome was performed to compare SNP genotypes of each sample that were called upon using reported draft genome and our assembled scaffolds as reference. Using LAST [38], we redefined the coordinate of our assembled scaffolds according to the reported draft genome. The following parameter options were adopted: ‘-c –m1111110’ and ‘-q3 –e35.’ We then isolated the loci that were exactly matched and calculated SNP genotype concordance for each sample.

### Phylogenomic analyses

The data matrix for the phylogenomic analyses consisted of four newly determined and 18 published mitochondrial genome sequences of baleen whales: 2 eschrichtiids, 14 balaenopterids, 2 neobalaenids, and 4 balaenids. Here, *Kogia breviceps* (Odontoceti, Kogiidae) was used as an outgroup. The mitochondrial genome sequences were initially aligned using MAFFT v6 [39] and then corrected by visual inspection. The final alignment included 16,435 nucleotides.

The phylogenomic analysis was carried out using the BI, ML, and NJ methods. We chose the best-fit model of nucleotide substitution with the standard ModelTest PAUP block in PAUP 4.0b10 [40] and Akaike’s information criterion (AIC) in ModelTest 3.7[41]; GTR + I + G was selected as the best evolutionary model. The uniformed BI analysis was implemented using MrBayes 3.2.1[42] with the GTR + I + G model. For the partitioned model approach of BI analysis, mitochondrial genomes were divided into 18 partitions and the following models were applied: GTR + I + G for the 12S rRNA, 16S rRNA, COX1, and 22 tRNAs regions; SYM + I for the 2 STS region; TVM + I + G for the NADH1, COX3, NADH4, and Control regions; HKY + G for the NADH2, ATPase8, and NADH4L regions; TVM + G for the COX2 region; HKY + I + G for the ATPase6 and NADH6 regions; TVM + I for the NADH3 region; K81uf + I + G for the NADH5 region; TrN + G for the Cytb region (Additional file 1: Table S12). Each analysis consisted of 20,000,000 generations with a burn-in of 20,000 and a sample frequency of 500. Bayesian posterior probability (BPP) values are shown on internal nodes to indicate the robustness of the phylogenomic analysis.

ML analysis was performed using PHYML 3.0[43] with a BIONJ starting tree under the GTR model and nonparametric bootstrap analysis was conducted with 500 pseudoreplicates. The proportion of invariable sites and gamma shape parameter were estimated from the dataset and the number of substitution rate categories was set to 6. The tree topology optimization was chosen.

NJ analysis [44] was conducted using the PHYLIP package 3.69 [45], based on Kimura’s [46] two-parameter distance. Ts/Tv ratios (10.10) were estimated from the data set using PUZZLE 4.0.2 [47] and then were used as inputs for the SEQBOOT, DNADIST, NEIGHBOUR, and CONSENS programs of the PHYLIP package. A bootstrap test (with 1,000 pseudoreplicates) [48] was performed to determine the statistical support for each node of the NJ tree.

### Co-estimation of evolutionary rates, TMRCA

To co-estimate the evolutionary rates and times to the most recent common ancestor (TMRCA), Bayesian coalescent approaches were implemented in BEAST 1.6.2 [49]. Crown Cetacea was calibrated based on the oldest mysticete fossil Llanocetus [50,51] (34 Mya, 35 mean, 1.0 SD. The age of the basal of the crown Mysticeti was calibrated based on an unnamed balaenid from New Zealand [52] (28 Mya, 29.0 mean, 1.0 SD). *Kogia breviceps* (Odontoceti; Kogiidae) was used as an outgroup. The analysis was conducted under the GTR + I + G model, nst = 6, and rates = gamma derived from AIC in ModelTest 3.7 [41]. We employed relaxed uncorrelated lognormal for molecular clock model and Yule process for tree topology prior. The data sets were each run for 20,000,000 generations to ensure convergence of all parameters (ESSs  >  200) with discarded burn-in of 10%. The resulting convergence was analyzed using Tracer 1.5 (http://beast.bio.ed.ac.uk/Tracer) and the statistical uncertainties were summarized in the 95% highest probability density (HPD) intervals. Trees were summarized as maximum clade credibility trees using the TreeAnnotator program, which forms part of the BEAST package, and were visualized using FigTree 1.4.0 (http://tree.bio.ed.ac.uk/software/figtree/).

### dN/dS ratio of orthologous genes

We used protein and reference cDNA sequences of human, mouse, dog, pig, cow, horse, and dolphin from Ensembl [52] and the common minke whale from our results. We used Hcluster_sg [53] to generate clusters based on BLAST 2.2.27 + [54] results. Then we generated multiple alignments for input into Mestortho [55] using PRANK31 [56]. Mestortho was used to identify the 1:1 orthologs of all eight species. As a result, 5,539 1:1 orthologs were identified and used to estimate the synonynous and nonsynonymous substitution rates. We obtained phylogenetic tree information from Timetree (www.timetree.org). Orthologous gene sets were aligned using PRANK31 [56], and poorly aligned sites were eliminated using Gblocks [57]. We used the ML method from codeml of PAML 4 [58] to estimate the dN (the rate of non-synonymous substitution), dS (the rate of synonymous substitution), and *ω* (the ratio of non-synonymous substitutions to the rate of synonymous substitutions) with F3X4 codon frequencies under the branch (model = 2, NSsites = 0) and basic models (model = 0, NSsites = 0). Results from the branch model were filtered with dS > 3 or dN/dS > 5. A log likelihood ratio test (LRT) was performed to compare these models; FDR adjustments for multiple testing corrections were applied [59], and a significance level of P < 0.05 was used.

### McDonald-Kreitman test

We used the protein and reference cDNA sequences of common minke whales and dolphins. We used the RBH method from BLAST 2.2.27 + [54]. As a result, 11,698 1:1 orthologs for the two species were identified. We generated multiple alignments using PRANK31 [56] and eliminated poorly aligned sites using Gblocks [57] before performing a standard McDonald-Kreitman test [22].

## Additional file

Additional file 1: Figure S1. Summary of read mapping to three assembled genomes using Bowtie2. **Figure S2.** BI tree using the partitioned model approach. Here the complete mitochondrial genome sequences were divided into 18 partitions. **Figure S3.** Biological Process of Gene Ontology of common minke whale showing 249 accelerated genes among 8 mammals. **Figure S4.** Overview of the assembly, gene prediction and variant calling process. **Figure S5.** Process of contig extention and bridging. **Table S1.** Summary statistics of common minke whale genome assembly by sample. **Table S2.** Summary of RepeatMasker results by sample. **Table S3.** Summary of gene prediction results using Augustus and Blastp. **Table S4.** Contig classification of the four samples. **Table S5.** Summary statistics of the combined common minke whale genome assembly. **Table S6.** Summary of repeat masking results using RepeatMask. **Table S7.** Summary of read mapping using Bowtie2. **Table S8.** Summary of variant calling results using GATK. **Table S9.** The result of SNP genotype concordance between using the reference of 1) reported draft genome and 2) assembled scaffolds of our study. **Table S10.** Species name of the sequences used in the present study with the GenBank accession numbers. New sequences obtained in this study are marked with an asterisk (*). **Table S11.** Sequencing results of the four common minke whale samples. **Table S12.** Best fitted model of each MT genomic region of the whales.
