# Supplementary material for: Cetaceans evolution: insights from the genome sequences of common minke whales
Source: BMC Genomics. 2015 Jan 22;16(1):13. doi: 10.1186/s12864-015-1213-1 (PMC4311506; doi:10.1186/s12864-015-1213-1)
Supplement: Additional file 1: Figure S1. — Summary of read mapping to three assembled genomes using Bowtie2. Figure S2. BI tree using the partitioned model approach. Here the complete mitochondrial genome sequences were divided into 18 partitions. Figure S3. Biological Process of Gene Ontology of common minke whale showing 249 accelerated genes among 8 mammals. Figure S4. Overview of the assembly, gene prediction and variant calling process. Figure S5. Process of contig extention and bridging. Table S1. Summary statistics of common minke whale genome assembly by sample. Table S2. Summary of RepeatMasker results by sample. Table S3. Summary of gene prediction results using Augustus and Blastp. Table S4. Contig classification of the four samples. Table S5. Summary statistics of the combined common minke whale genome assembly. Table S6. Summary of repeat masking results using RepeatMask. Table S7. Summary of read mapping using Bowtie2. Table S8. Summary of variant calling results using GATK. Table S9. The result of SNP genotype concordance between using the reference of 1) reported draft genome and 2) assembled scaffolds of our study. Table S10. Species name of the sequences used in the present study with the GenBank accession numbers. New sequences obtained in this study are marked with an asterisk (*). Table S11. Sequencing results of the four common minke whale samples. Table S12. Best fitted model of each MT genomic region of the whales. [file 12864_2015_1213_MOESM1_ESM.docx]

| SUPPLEMENTARY INFORMATION |
| --- |

**Cetacean evolution: insights from the genome sequences of common minke whales**

Jung Youn Park, Yong-Rock An, Naohisa Kanda, Chul-Min An, Hye-suk An, Jung-Ha Kang, Eun Mi Kim, Du-Hae An, Hojin Jung, Myunghee Joung, Myung Hum Park, Sook Hee Yoon, Bo-Young Lee, Taeheon Lee, Kyu-Won Kim, WonCheoul Park, DongHyun Shin, YoungSub Lee, Jaemin Kim, Woori Kwak, Hyeon Jeong Kim, Young-Jun Kwon, Sunjin Moon, Yuseob Kim, David W Burt, Seoae Cho, Heebal Kim

| **TABLE OF CONTENTS** |
| --- |

1. **Supplementary Figures …………………………………………………p.3**
   1. **Supplementary Figure 1. ………………………………………p.3**
   2. **Supplementary Figure 2. ………………………………………p.4**
   3. **Supplementary Figure 3. ………………………………………p.5**
   4. **Supplementary Figure 4. ………………………………………p.6**
   5. **Supplementary Figure 5. ………………………………………p.7**
2. **Supplementary Tables……………………………………………………p.8**
   1. **Supplementary Table 1. ………………………………………..p.8**
   2. **Supplementary Table 2. ………………………………………..p.9**
   3. **Supplementary Table 3. ………………………………………..p.10**
   4. **Supplementary Table 4. ………………………………………..p.11**
   5. **Supplementary Table 5. ………………………………………..p.12**
   6. **Supplementary Table 6. ………………………………………..p.13**
   7. **Supplementary Table 7. ………………………………………..p.14**
   8. **Supplementary Table 8. ………………………………………..p.15**
   9. **Supplementary Table 9. ………………………………………..p.16**
   10. **Supplementary Table 10. ………………………………………p.17**
   11. **Supplementary Table 11. …………………………………….....p.18**
   12. **Supplementary Table 12. …………………………………….....p.19**


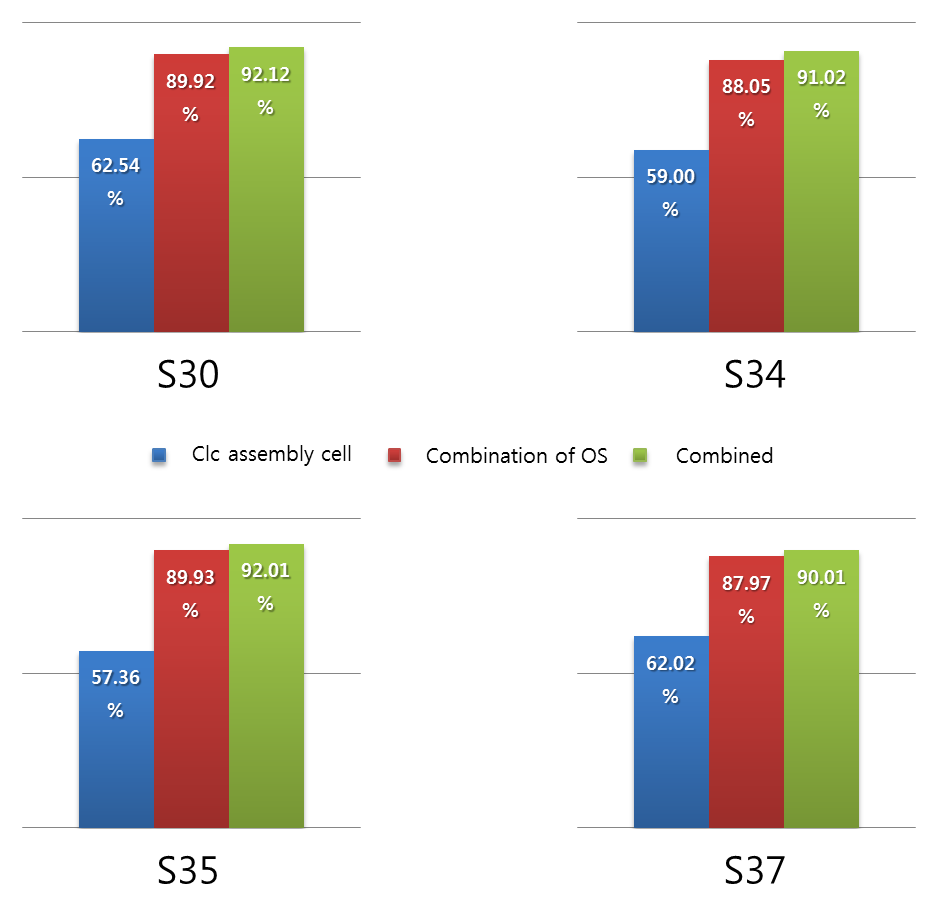


**Supplementary Figure 1.** Summary of read mapping to three assembled genomes using Bowtie2.

**Supplementary Figure 2.** BI tree using the partitioned model approach. Here the complete mitochondrial genome sequences were divided into 18 partitions.


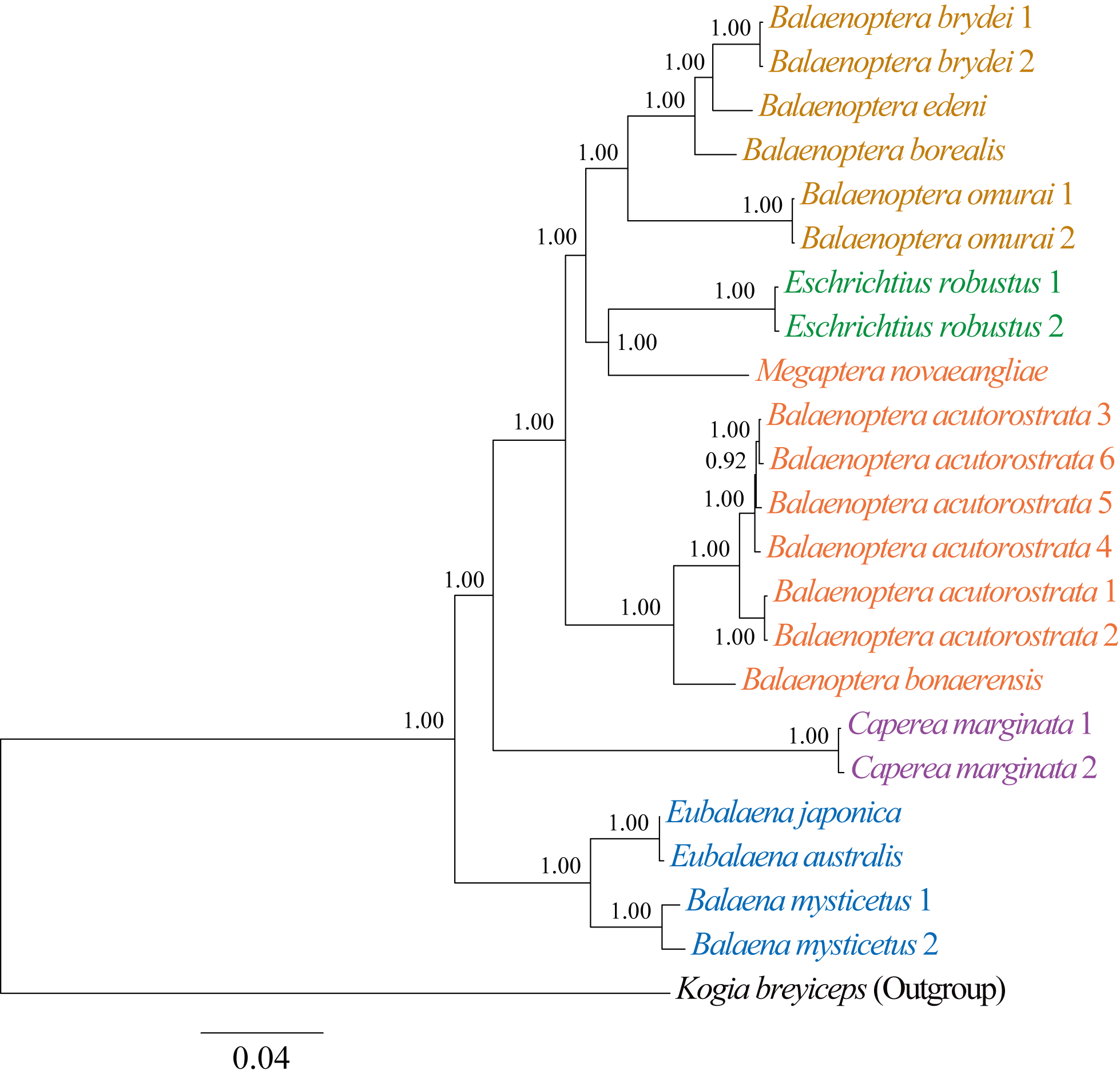


**Supplementary Figure 3.** Biological Process of Gene Ontology of common minke whale showing 249 accelerated genes among 8 mammals

**

**


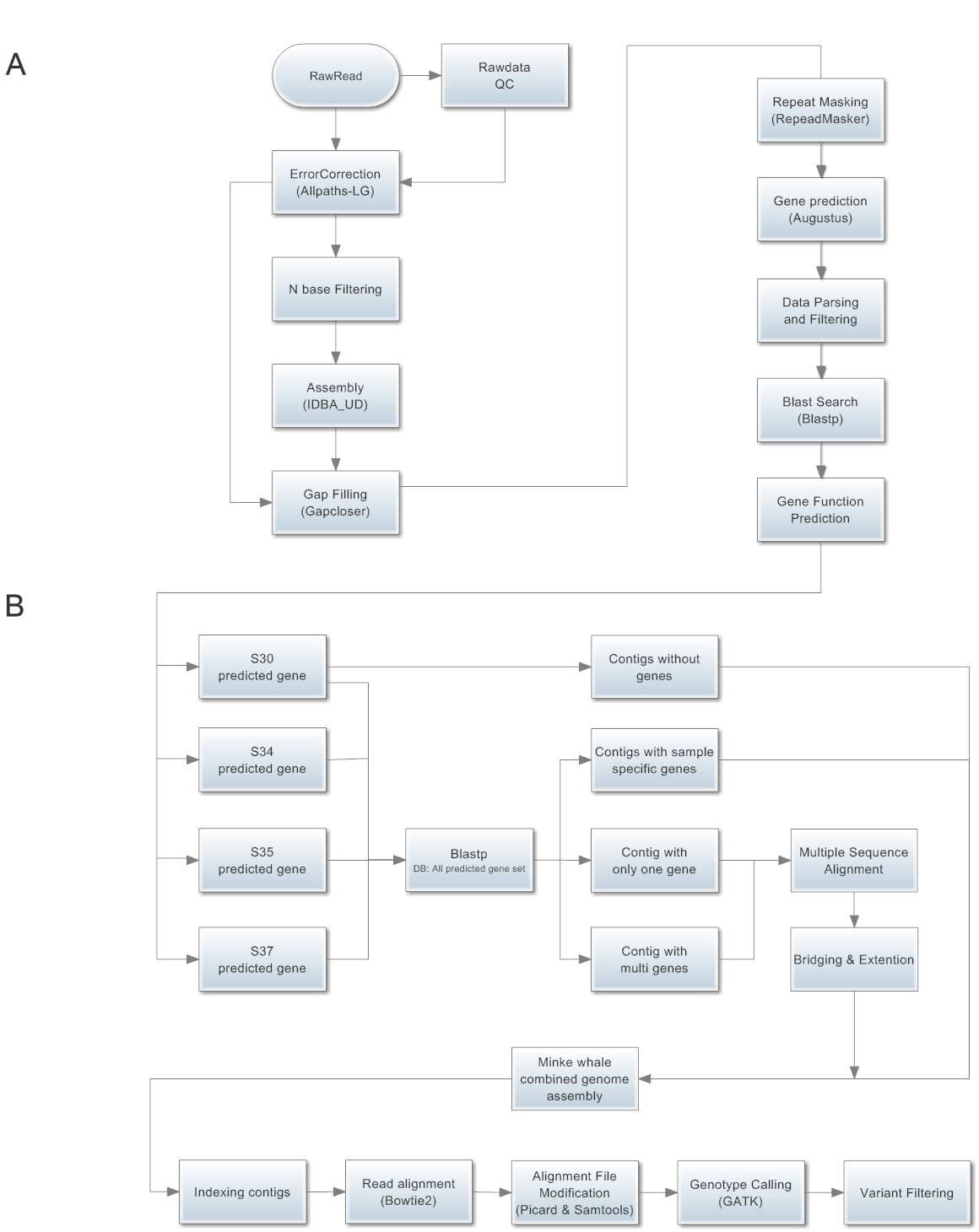


**Supplementary Figure 4**. Overview of the assembly, gene prediction and variant calling process.

A. Genome assembly and gene prediction process. B. Assembly building process.


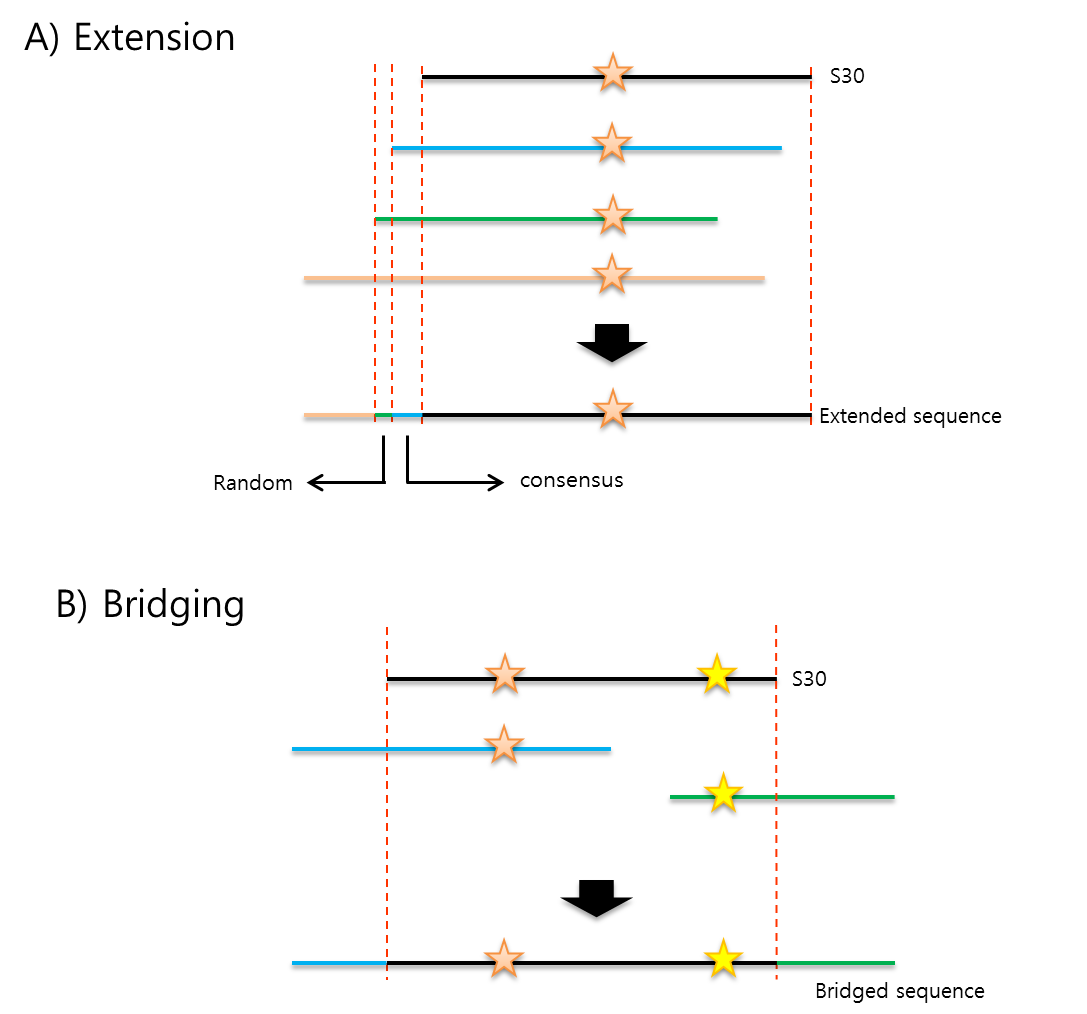


**Supplementary Figure 5**. Process of contig extention and bridging.

1. Process of Extension. The star shows the same gene between clusters.
2. Process of bridging. The stars show multiple genes in the S30 contig.
3. **Supplementary Tables**

**Supplementary Table 1**. Summary statistics of common minke whale genome assembly by sample.

| **Sample name** | S30 | S34 | S35 | S37 |
| --- | --- | --- | --- | --- |
| **Number of contigs** | 262,747 | 313,490 | 282,736 | 294,439 |
| **Sequence length** |  |  |  |  |
| Minimum length | 2,000 | 2,000 | 2,000 | 2,000 |
| Maximum length | 105,339 | 61,954 | 53,070 | 68,378 |
| Average length | 7,651 | 4,719 | 3,985 | 6,286 |
| N50 length | 10,321 | 5,359 | 4,236 | 7,810 |
| **Residue information** |  |  |  |  |
| Total residue count (bp) | 2,010,222,571 | 1,479,651,607 | 1,126,905,396 | 1,851,132,035 |
| N contents | 17,875 | 303,142 | 4,484,941 | 2,988,329 |
| Closed N by Gapcloser | 15,243 | 239,309 | 727,324 | 1,298,712 |
| GC content (%) | 40.51 | 38.58 | 43.21 | 40.59 |

*Considered contigs with length > 2,000 bp

**Supplementary Table 2**. Summary of RepeatMasker results by sample.

| **Sample name** | **S30** | | **S34** | | **S35** | | **S37** | |
| --- | --- | --- | --- | --- | --- | --- | --- | --- |
| **Elements** | **No.** | **Length** | **No.** | **Length** | **No.** | **Length** | **No.** | **Length** |
| Sine | 1,015,281 | 150,800,833 (6.77%) | 742,339 | 105,477,583  (6.31%) | 123,993 | 17,666,179  (1.66%) | 861,733 | 125,495,334  (6.01%) |
| Line | 1,280,241 | 456,915,337  (20.52%) | 1,017,906 | 344,636,222  (20.61%) | 186,869 | 59,040,424  (5.54%) | 1,203,118 | 415,185,547  (19.87%) |
| LTR elements | 455,514 | 144,478,462  (6.49%) | 369,818 | 110,307,049  (6.60%) | 74,671 | 24,543,226  (2.30%) | 435,092 | 139,417,009  (6.67%) |
| DNA elements | 369,942 | 78,295,071  (3.52%) | 292,067 | 60,486,743  (3.62%) | 56,988 | 11,114,767  (1.04%) | 358,544 | 74,122,957  (3.55%) |
| Unclassified | 5,055 | 956,831  (0.04%) | 4,024 | 751,816  (0.04%) | 771 | 134,971  (0.01%) | 4,968 | 927,640  (0.04%) |
| Small RNA | 5,272 | 575,413 (0.03%) | 3,936 | 429,088  (0.03%) | 787 | 84,733  (0.01%) | 4,931 | 547,234  (0.03%) |
| Satellites | 183,837 | 62,416,759  (2.80%) | 140,168 | 46,115,494  (2.76%) | 29,359 | 10,183,045  (0.96%) | 169,308 | 55,462,818  (2.65%) |

**Supplementary Table 3**. Summary of gene prediction results using Augustus and Blastp.

|  | **S30** | **S34** | **S35** | **S37** |
| --- | --- | --- | --- | --- |
| Number of genes | 41,098 | 28,348 | 30,383 | 37,422 |
| Number of Exons | 131,164 | 62,167 | 73,950 | 113,794 |
| Total gene length | 229,649,018 | 82,573,850 | 84,286,704 | 178,879,019 |
| Average length | 5587.84 | 2912.86 | 2774.14 | 4780.05 |

* Over 100 peptide length and 70% gene coverage.

**Supplementary Table 4**. Contig classification of the four samples.

| **Source** | **S30** | **S35** | **S34** | **S37** |
| --- | --- | --- | --- | --- |
| Number of contigs | 628,081 | 938,541 | 994,603 | 668,188 |
| Number of genes | 41,098 | 30,383 | 28,348 | 37,422 |
| Contig with genes | 27,635 | 18,268 | 14,849 | 24,506 |
| Contig with one gene | 14,285 | 11,065 | 8,057 | 14,296 |
| Contig with multi genes | 600 | 542 | 212 | 637 |
| Contig with sample specific gene | 12,750 | 6,661 | 6,580 | 9,573 |
| Contig without gene | 600,446 | 920,273 | 979,754 | 643,682 |

**Supplementary Table 5**. Summary statistics of the combined common minke whale genome assembly.

| **Source** | **Value** |
| --- | --- |
| **Number of Contigs** | 286,129 |
| **Sequence Length** |  |
| Minimum length | 2,000 |
| Maximum length | 105,339 |
| Average length | 7,727 |
| N50 length | 10,400 |
| **Residue information** |  |
| Total residue count (bp) | 2,211,014,055 |
| N content | 524,763(0.02%) |
| GC content (%) | 40.92% |

*Considered contigs with length > 2,000 bp

**Supplementary Table 6**. Summary of repeat masking results using RepeatMask.

| **Source** | **Number of elements** | **Length occupied (%)** |
| --- | --- | --- |
| Total length | - | 2,226,669,555 |
| SINE | 1,015,281 | 150,800,833  (6.77%) |
| LINE | 1,280,241 | 456,915,337  (20.52%) |
| LTR elements | 455,514 | 144,478,462  (6.49%) |
| DNA elements | 369,942 | 78,295,071  (3.52%) |
| Unclassified | 5,055 | 956,831  (0.04%) |
| Small RNA | 5,272 | 575,413  (0.03%) |
| Satellites | 183,837 | 62,416,759  (2.80%) |
| Total Masked |  | 893,922,896 (40.15%) |

**Supplementary Table 7**. Summary of read mapping using Bowtie2.

| **Categories** | **Samples** | | | |
| --- | --- | --- | --- | --- |
|  | **30** | **34** | **35** | **37** |
| Total number of reads | 514,451,848  (100%) | 402,081,180  (100%) | 470,186,674  (100%) | 402,636,650  (100%) |
| Concordantly 1 time | 290,495,048  (56.4%) | 211,464,096  (52.5%) | 236,048,842  (50.2%) | 162,187,448  (40.2%) |
| Concordantly > 1 time | 85,699,662  (16.6%) | 76,771,800  (19.0%) | 79,939,174  (17.0%) | 34,242,644  (8.5%) |
| Discordantly 1 time | 14,322,176  (2.7%) | 6,252,922  (1.5%) | 19,396,744  (4.1%) | 55,572,032  (13.8%) |
| 1 time in mixed mode (single reads) | 40,957,298  (7.9%) | 33,725,380  (8.3%) | 33,216,429  (7.0%) | 49,889,813  (12.3%) |
| > 1 time in mixed mode (single reads) | 31,097,230  (6.0%) | 25,804,959  (6.4%) | 54,233,679  (11.5%) | 52,303,191  (12.9%) |
| Overall mapping rate including singletons | 462,571,414  (89.9%) | 354,019,157  (88.0%) | 422,834,868  (89.9%) | 354,195,128  (87.9%) |

**Supplementary Table 8**. Summary of variant calling results using GATK.

|  | **Categories** | **S30** | **S34** | **S35** | **S37** | **Total** |
| --- | --- | --- | --- | --- | --- | --- |
| INDEL | Raw | 306,761  (14,727) | 410,849  (32,206) | 370,877  (21,780) | 386,011  (20,128) | 554,937 |
|  | Pass | 214,531  (10,635) | 309,103  (28,352) | 277,948  (18,574) | 290,629  (18,434) | 414,932 |
|  | Missing filter | 198,957  (9,941) | 288,353  (25,443) | 272,358  (18,359) | 275,062  (17,744) | 389,542 |
| SNV | Raw | 2,482,691  (213,821) | 3,266,565  (429,416) | 3,188,965  (395,933) | 3,225,149  (383,228) | 5,137,672 |
|  | Pass SNV | 1,880,150  (184,309) | 2,625,283  (388,023) | 2,479,768  (341,658) | 2,543,039  (353,259) | 4,101,990 |
|  | Biallele filter | 1,879,332  (184,309) | 2,624,524  (388,016) | 2,478,951  (341,646) | 2,542,204  (353,246) | 4,099,489 |
|  | Missing filter | 1,673,581  (162,462) | 2,390,171  (352,689) | 2,344,185  (325,738) | 2,361,319  (333,604) | 3,730,122 |

* In the rows showing INDELs and SNPs, the number of sample specific variants are enclosed in parentheses

**Supplementary Table 9**. The result of SNP genotype concordance between using the reference of 1) reported draft genome and 2) assembled scaffolds of our study.

|  | S30 | S32 | S34 | S37 |
| --- | --- | --- | --- | --- |
| Number of loci | 529,793 | 581,468 | 546,596 | 542,952 |
| Matched genotype | 523,353 | 570,191 | 535,412 | 526,618 |
| Genotype  Concordance | 98.78% | 98.06% | 97.95% | 96.99% |

**Supplementary Table 10**. Species name of the sequences used in the present study with the GenBank accession numbers. New sequences obtained in this study are marked with an asterisk (*).

| Classification | Species | Accession no. |
| --- | --- | --- |
| 1. Mysticeti; Balaenidae | *Balaena mysticetus* 1 | AP006472 |
| 1. Mysticeti; Balaenidae | *Balaena mysticetus* 2 | NC_005268 |
| 1. Mysticeti; Balaenidae | *Eubalaena australis* | NC_006930 |
| 1. Mysticeti; Balaenidae | *Eubalaena japonica* | NC_006931 |
| 1. Mysticeti; Balaenopteridae | *Balaenoptera acutorostrata* 1 | AP006468 |
| 1. Mysticeti; Balaenopteridae | *Balaenoptera acutorostrata* 2 | NC_005271 |
| 1. Mysticeti; Balaenopteridae | *Balaenoptera acutorostrata* 3 | *Korea 30 |
| 1. Mysticeti; Balaenopteridae | *Balaenoptera acutorostrata* 4 | *Korea 34 |
| 1. Mysticeti; Balaenopteridae | *Balaenoptera acutorostrata* 5 | *Korea 35 |
| 1. Mysticeti; Balaenopteridae | *Balaenoptera acutorostrata* 6 | *Korea37 |
| 1. Mysticeti; Balaenopteridae | *Balaenoptera bonaerensis* | NC_006926 |
| 1. Mysticeti; Balaenopteridae | *Balaenoptera borealis* | NC_006929 |
| 1. Mysticeti; Balaenopteridae | *Balaenoptera brydei* 1 | AB201259 |
| 1. Mysticeti; Balaenopteridae | *Balaenoptera brydei* 2 | NC_006928 |
| 1. Mysticeti; Balaenopteridae | *Balaenoptera edeni* | NC_007938 |
| 1. Mysticeti; Balaenopteridae | *Balaenoptera omurai* 1 | AB201257 |
| 1. Mysticeti; Balaenopteridae | *Balaenoptera omurai* 2 | NC_007937 |
| 1. Mysticeti; Balaenopteridae | *Megaptera novaeangliae* | NC_006927 |
| 1. Mysticeti; Neobalaenidae | *Caperea marginata* 1 | AP006475 |
| 1. Mysticeti; Neobalaenidae | *Caperea marginata* 2 | NC_005269 |
| 1. Mysticeti; Eschrichtiidae | *Eschrichtius robustus* 1 | AP006471 |
| 1. Mysticeti; Eschrichtiidae | *Eschrichtius robustus* 2 | NC_005270 |
| 1. Odontoceti; Kogiidae | *Kogia breviceps* | NC_005272 |

**Supplementary Table 11**. Sequencing results of the four common minke whale samples.

| Sample Name | Insert  Size | Total Base (bp) | Depth (X) | Read Count | N (%) | GC (%) | Q20 ratio(%) /depth(X) | Q30 ratio(%) /depth(X) |
| --- | --- | --- | --- | --- | --- | --- | --- | --- |
| S30 | 270bp | 51,959,636,648 | 17.32 | 514,451,848 | 1.98 | 39.89 | 92 / 15.9 | 87 / 15.0 |
| S34 | 270bp | 40,610,199,180 | 13.54 | 402,081,180 | 2.54 | 39.72 | 92 / 12.3 | 87 / 11.8 |
| S35 | 480bp | 47,488,854,074 | 15.83 | 470,186,674 | 1.82 | 42.4 | 90 / 14.2 | 83 / 13.1 |
| S37 | 480bp | 40,666,301,650 | 13.56 | 402,636,650 | 2.17 | 40.98 | 89 / 12.1 | 82 / 11.1 |
| Total | | 180,724,991,552 | 60.25 | 1,789,356,352 | 2.13 | 40.75 | 90 / 54.5 | 85 / 51.0 |

* Estimated common minke whale genome size : 3 Gb

* Fastq Quality Encoding : Sanger Quality ( ASCII Character Code = Phred Quality Value + 33 )

**Supplementary Table 12.** Best fitted model of each MT genomic region of the whales.

| Genomic region | Model |
| --- | --- |
| 1. 12S rRNA | GTR+I+G, Nst=6, Rates=gamma |
| 2. 16S rRNA | GTR+I+G, Nst=6, Rates=gamma |
| 3. 2 STS | SYM+I, Nst=6, Rates=equal |
| 4. NADH1 | TVM+I+G, Nst=6, Rates=gamma |
| 5. NADH2 | HKY+G, Nst=2, Rates=gamma |
| 6. COX1 | GTR+I+G, Nst=6, Rates=gamma |
| 7. COX2 | TVM+G, Nst=6, Rates=gamma |
| 8. ATPase8 | HKY+G, Nst=2, Rates=gamma |
| 9. ATPase6 | HKY+I+G, Nst=2, Rates=gamma |
| 10. COX3 | TVM+I+G, Nst=6, Rates=gamma |
| 11. NADH3 | TVM+I, Nst=6, Rates=equal |
| 12. NADH4L | HKY+G, Nst=2, Rates=gamma |
| 13. NADH4 | TVM+I+G, Nst=6, Rates=gamma |
| 14. NADH5 | K81uf+I+G, Nst=6, Rates=gamma |
| 15. NADH6 | HKY+I+G, Nst=2, Rates=gamma |
| 16. Cytb | TrN+G, Nst=6, Rates=gamma |
| 17. Control region | TVM+I+G, Nst=6, Rates=gamma |
| 18. 22 tRNAs | GTR+I+G, Nst=6, Rates=gamma |
